# DNA Damage Response Proteins and Oxygen Modulate Prostaglandin E_2_ Growth Factor Release in Response to Low and High LET Ionizing Radiation

**DOI:** 10.3389/fonc.2015.00260

**Published:** 2015-12-07

**Authors:** Christopher P. Allen, Walter Tinganelli, Neelam Sharma, Jingyi Nie, Cory Sicard, Francesco Natale, Maurice King, Steven B. Keysar, Antonio Jimeno, Yoshiya Furusawa, Ryuichi Okayasu, Akira Fujimori, Marco Durante, Jac A. Nickoloff

**Affiliations:** ^1^Department of Environmental and Radiological Health Sciences, Colorado State University, Fort Collins, CO, USA; ^2^GSI Helmholtzzentrum für Schwerionenforschung GmbH, Darmstadt, Germany; ^3^Research Development and Support Center, National Institute of Radiological Sciences, Chiba, Japan; ^4^Division of Medical Oncology, University of Colorado School of Medicine, Aurora, CO, USA; ^5^Research Center for Radiation Protection, National Institute of Radiological Sciences, Chiba, Japan; ^6^Research Center for Charged Particle Therapy, National Institute of Radiological Sciences, Chiba, Japan

**Keywords:** radiotherapy, DNA damage response, growth factor, apoptosis, caspase

## Abstract

Common cancer therapies employ chemicals or radiation that damage DNA. Cancer and normal cells respond to DNA damage by activating complex networks of DNA damage sensor, signal transducer, and effector proteins that arrest cell cycle progression, and repair damaged DNA. If damage is severe enough, the DNA damage response (DDR) triggers programed cell death by apoptosis or other pathways. Caspase 3 is a protease that is activated upon damage and triggers apoptosis, and production of prostaglandin E_2_ (PGE_2_), a potent growth factor that can enhance growth of surviving cancer cells leading to accelerated tumor repopulation. Thus, dying tumor cells can promote growth of surviving tumor cells, a pathway aptly named Phoenix Rising. In the present study, we surveyed Phoenix Rising responses in a variety of normal and established cancer cell lines, and in cancer cell lines freshly derived from patients. We demonstrate that IR induces a Phoenix Rising response in many, but not all cell lines, and that PGE_2_ production generally correlates with enhanced growth of cells that survive irradiation, and of unirradiated cells co-cultured with irradiated cells. We show that PGE_2_ production is stimulated by low and high LET ionizing radiation, and can be enhanced or suppressed by inhibitors of key DDR proteins. PGE_2_ is produced downstream of caspase 3 and the cyclooxygenases COX1 and COX2, and we show that the pan COX1–2 inhibitor indomethacin blocks IR-induced PGE_2_ production in the presence or absence of DDR inhibitors. COX1–2 require oxygen for catalytic activity, and we further show that PGE_2_ production is markedly suppressed in cells cultured under low (1%) oxygen concentration. Thus, Phoenix Rising is most likely to cause repopulation of tumors with relatively high oxygen, but not in hypoxic tumors. This survey lays a foundation for future studies to further define tumor responses to radiation and inhibitors of the DDR and Phoenix Rising to enhance the efficacy of radiotherapy with the ultimate goal of precision medicine informed by deep understanding of specific tumor responses to radiation and adjunct chemotherapy targeting key factors in the DDR and Phoenix Rising pathways.

## Introduction

**Figure 1:**
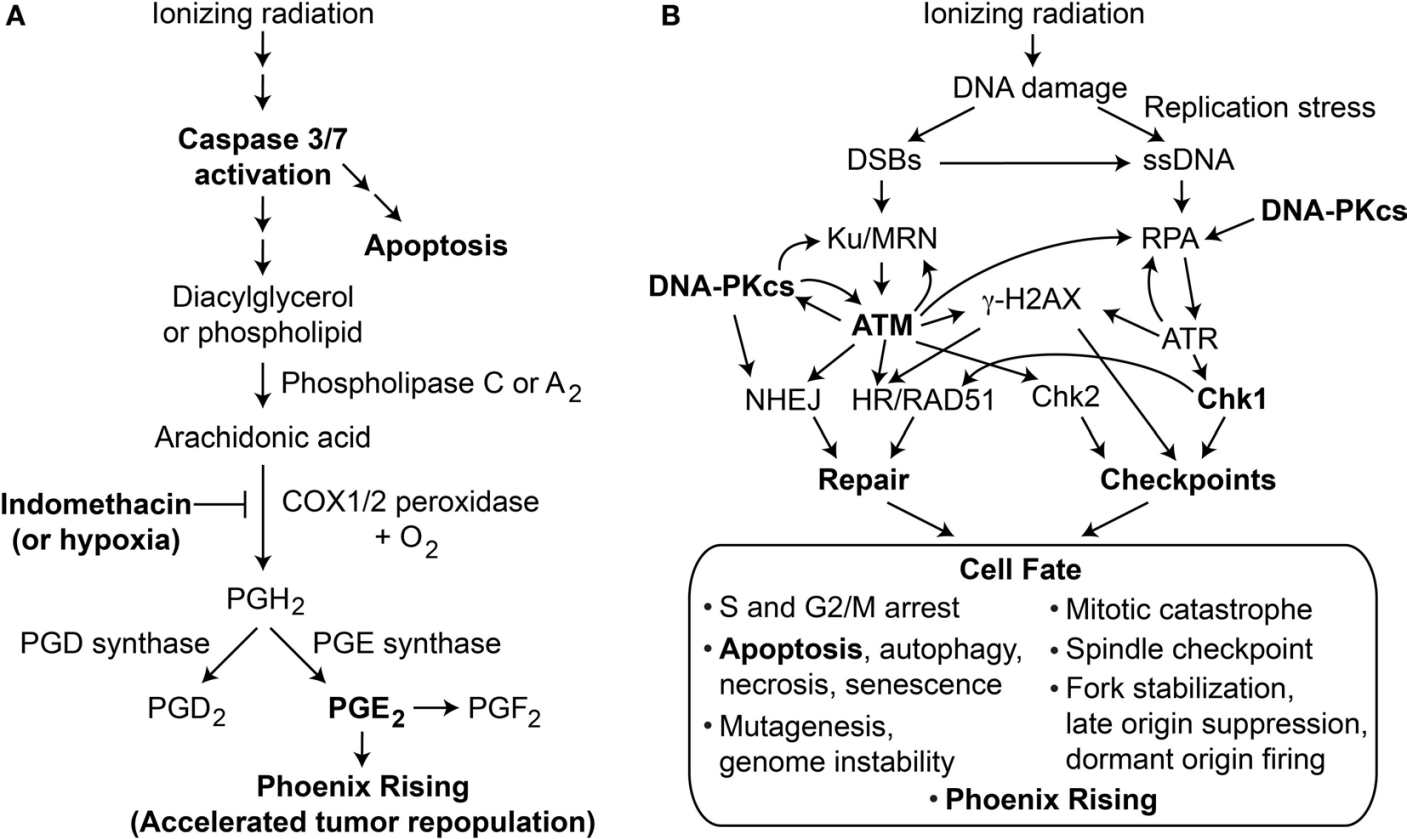
**(A)** Phoenix Rising pathway of accelerated tumor repopulation. The cascade is initiated by cleavage and activation of caspase 3, which also promotes programed cell death by apoptosis. Activated caspase 3 cleaves and activates phospholipase 2 that hydrolyzes fatty acid phospholipid bonds, releasing arachidonic acid and lysophospholipids. Arachidonic acid is converted to PGH_2_ by COX1 and COX2 peroxidases in the presence of oxygen; this step can be blocked by COX1–COX2 inhibitor indomethacin or hypoxia. Prostaglandin synthases generate the family of prostanoids, including PGD_2_, PGE_2_, and PGF_2_. PGE_2_ (and possibly other PGs) excreted from dying cells promote growth of surviving cells, accelerating tumor repopulation. **(B)** The DDR regulates cell fate after radiation damage. Proteins involved in DNA repair and damage checkpoint pathways crosstalk with programed cell death pathways to determine a variety of short- and long-term cell fates. Phoenix Rising and the DDR are linked through apoptosis and possibly other processes.

The majority of cancer patients receive radiotherapy (RT), and virtually all cancer treatments employ chemical or physical genotoxins that directly damage DNA, or inhibit DNA metabolism (such as topoisomerase inhibitors). DNA damage activates DNA damage response (DDR) pathways, but the specific DDR pathways activated, the degree of activation, and cell fate depend on many factors, including the amount and type of damage, as well as the genetic and environmental state of the cell (cell type, cell cycle phase, normal vs. tumor, hypoxic vs. normoxic, etc). Low and high LET IR yield different dose distributions in tissue and induce different types of damage, and may, thus, differentially activate DDR pathways. Solid tumors are genetically heterogeneous, which contributes to therapeutic resistance (1), and it is difficult to achieve 100% elimination of tumor cells while minimizing normal tissue toxicity. Rare surviving tumor cells, thus, pose a risk of tumor repopulation. A long recognized problem is that following RT, tumors may be rapidly repopulated, a phenomenon termed “accelerated tumor repopulation.” The Li lab identified a paracrine growth factor signaling pathway that contributes to accelerated repopulation called “Phoenix Rising” (2–4). This pathway is initiated when lethally irradiated tumor cells activate caspase 3/7 (a key step in caspase-dependent apoptosis), leading to production of prostaglandin E^2^ (PGE^2^), a potent growth factor (Figure 1A). Thus, dying cells trigger growth of neighboring viable cells, a process akin to wound healing. PGE_2_ produced via Phoenix Rising stimulates cell growth in culture and tumor repopulation in mice (2). Given the high radiation doses required to kill high fractions of tumor cells, the rare surviving cells are likely to experience significant DNA damage, and rapid proliferation of such cells is expected to enhance mutagenesis and may drive tumor progression toward a more aggressive metastatic state. There is accumulating evidence that PGE_2_/Phoenix Rising is clinically relevant. Patients with head and neck squamous cell carcinoma or breast cancers that express caspase 3 show reduced survival (2), PGE_2_ promotes renal carcinoma cell invasion that may contribute to metastasis (5), and recent studies indicate that blocking PGE_2_ production with cyclooxygenase inhibitors improves outcomes in bladder and breast cancer patients treated with chemotherapy or RT (6, 7).

The DDR comprises complex networks of DNA repair and DNA damage signaling (checkpoint) pathways that control cell fate in response to DNA damage; a simplified view of DDR responses to ionizing radiation is shown in Figure 1B. The most fundamental cell fate decision is survival vs. death. Central to the DDR are protein kinases, including upstream PI3-like kinases ATM, ATR, and DNA-PKcs, that converge on downstream checkpoint kinases Chk1 and Chk2 (8). When cells experience limited damage, these factors promote cell survival and suppress cancer by effecting cell cycle arrest, stimulating repair, and promoting genome stability. Above a certain DNA damage threshold, these pathways promote senescence or programed cell death by apoptosis, necrosis, or autophagy (9, 10). Thus, DDR pathways are not “on or off” but show graded responses depending on the level of damage, and DDR thresholds are known to be genetically regulated (11, 12), and may vary for each checkpoint (13). There is substantial crosstalk among checkpoint and DNA repair pathways (14–22), and a major goal in the field is to identify synthetic (genetic) lethal interactions to exploit in cancer therapy (23).

There is considerable interest in targeting DDR proteins to augment therapeutic responses to chemotherapy and/or RT (24–26), for example, by sensitizing tumor cells to DNA damage. A common goal in cancer therapy is to kill tumor cells by inducing apoptosis. However, increasing caspase 3-dependent apoptosis may be a double-edged sword, leading initially to increased tumor killing, but accompanied by increased PGE_2_ secretion and subsequent growth stimulation of rare surviving tumor cells. The present study was designed to determine whether modulating the DDR (by chemical inhibition of DNA-PKcs, ATM, or Chk1) influences Phoenix Rising in a variety of normal or tumor cells following exposure to low and high LET IR. Phoenix Rising can be blocked at many steps along the pathway from caspase-3 cleavage, to PGE_2_ production/receptor binding (2, 27), and we tested whether chemical inhibition of COX1 and COX2 (COX1–2) blocked PGE_2_ production. Certain tumors are hypoxic, and because oxygen is a necessary co-factor for COX1–2 activity, we also tested whether PGE_2_ production differed under normoxic vs. hypoxic conditions. We show that PGE_2_ production, and proliferation of co-cultured unirradiated cells vary widely among cell lines. In some cell lines, we observed enhanced PGE_2_ production with inhibition of DNA-PKcs, suppression by inhibition of ATM, and both COX1–2 inhibition and hypoxia robustly suppressed PGE_2_ production. In general, PGE_2_ production did not affect short-term growth of irradiated cells (up to 48 h post IR), but PGE_2_ levels correlated with growth of co-cultured, unirradiated cells in longer-term growth assays. Interestingly, both oxygen concentration and LET alter PGE_2_ production. Together, these findings suggest that RT of certain tumor types may be enhanced by specific combinations of DDR and/or COX1–2 inhibitors that enhance tumor cell killing and mitigate accelerated tumor repopulation.

## Materials and Methods

### Cell Culture and Chemical Inhibitors

Human cell lines HeLa, HT1080, HCT116, MCF7, BJ1hTERT, and HFL3, and mouse melanoma D17 cells, were cultured in Dulbecco’s minimal essential medium (DMEM, Gibco) with 10% fetal bovine serum (Sigma or Atlas Biologicals), 100 IU/mL penicillin, 100 μg/mL streptomycin, 2.5 μg/mL amphotericin B (antibiotic/antimycotic, LifeTechnologies), 1 mM sodium pyruvate (Gibco) and incubated at 37°C with 5% CO_2_ in air. For the hypoxia experiments, HeLa cells were maintained in a hypoxic incubator in the same media and growth conditions except that the oxygen concentration was limited to 1%. Primary head and neck tumor [patient-derived xenograft (PDX)] cell lines CUHN013, CUHN036 (28), CUHN065, and CUHN067 were cultured in Rhesus Monkey Kidney, *Mucaca mulatta* (RM_K_) primary cell line media consisting of DMEM:F12 (3:1) with 10% FBS, insulin (5 μg/mL), hEGF (10 ng/mL), hydrocortisone (0.4 μg/mL), transferrin (5 μg/mL), penicillin (200 units/mL), and streptomycin (200 μg/mL).

Inhibitors of ATM (KU55933), Chk1 (UCN-01) DNA-PKcs (NU7026), and COX1–2 [indomethacin (Indo)] were purchased from Tocris Bioscience or Sigma and stored in powdered form at −20 or 4°C (NU7026). All compounds were freshly solubilized in DMSO to 100× working concentrations immediately prior to addition to cell cultures. Master mixes containing 1× final concentration of inhibitors in fresh media were prepared and added to wells pre- and post-irradiation. Final inhibitor concentrations were: 10 μM for ATMi, DNA-PKi, and COX1–2i, and 100 nM for Chk1i.

### Human-Derived Head and Neck Squamous Cell Carcinoma Cell Lines

Head and neck squamous cell carcinoma patients were consented at the University of Colorado Hospital in accordance with the protocol approved by the Colorado Multiple Institutional Review Board (COMIRB #: 08-0552). CUHN013, CUHN065, and CUHN067 cell lines were derived directly from fresh patient post-surgical tumor tissue. Due to minimal tissue procured, the CUHN036 cell line required expansion and was, therefore, derived from PDX tumors. Tumor tissue was processed into ~2 mm × 2 mm × 2 mm pieces using a scalpel and forceps and two to three pieces were placed in wells of cell culture grade six-well dishes without media. Uncovered plates were placed in the back of a cell culture hood and tumor pieces were allowed to dry/adhere to the plate for 15 min, then 2 mL of RM_K_ media was added to each well. Fresh media was added to tumor slices twice per week.

Outgrowing cells were characterized by flow cytometry (Cyan-ADP, Beckman Coulter) to confirm the presence of epithelial cancer cells (anti-CD44-APC, anti-EPCAM-FITC, anti-EGFR-PE) within the cancer-associated fibroblast cells (anti-mouse H2kd-PerCP–Cy5.5 for PDX tissue). Once cell populations had expanded sufficiently (~10^7^ cells), cells were sorted (MoFlo-XDP, Beckman Coulter) twice in succession using the above combination of cell surface markers to eliminate contaminating fibroblasts. To confirm the origin of resulting cell lines, we conducted short tandem repeat (STR) analysis comparing sorted cells to the originating patient tissue. Finally, tumors generated in immune-compromised nude mice from these human-derived cell lines recapitulated the morphology and histology of the original patient or PDX tumors.

### PGE_2_ Detection by ELISA

Cells (10,000–20,000) were seeded into individual wells of 96-well microtiter dishes and incubated overnight using two to three replicate wells per treatment group. The dishes were irradiated with 10 Gy γ-rays (CSU, ^137^Cs source), or 3 or 10 Gy X-rays (NIRS) low LET IR. The cells were treated with either DDR or COX-1/COX-2 inhibitors 12–16 h prior to IR and the inhibitors were present in the media during and after IR. PGE_2_ concentrations in growth media were measured at 0, 24, and 48 h after IR using a PGE_2_ Parameter ELISA kit (R & D Systems) according to the manufacturer’s directions. PGE_2_ standard concentration curves (Figure S1 in Supplementary Material) were derived from dilutions of pure PGE_2_ (R & D Systems) and fit to asymmetric 5-parameter logistic non-linear regressions using Prism software (Graphpad).

### Cell Proliferation Assay

Cell proliferation was measured using sulforhodamine B (SRB) assays (29) performed on cells adhering to PGE_2_ assay plates, since PGE_2_ assays required only the growth media, and SRB assays required only adherent cells. Optical densities were measured at 560 nm wavelength in a 96-well microtiter plate reader and baseline readings for controls (empty wells and media only wells) were subtracted to yield final O.D. values. The resultant data were processed using Excel and Prism software.

### PGE_2_ Detection by Liquid Chromatography-Tandem Mass Spectrometry

BJ1hTERT or HT1080 cells (300,000) were seeded into T-25 flasks, incubated overnight and pretreated with DDR inhibitors as above. Supernatants from non-irradiated samples and samples irradiated with 3 Gy high LET (70 keV/μm) carbon ion IR were collected 48 h post irradiation and stabilized by the addition of 0.1% (v/v) butylated hydroxytoluene. The samples were immediately frozen, stored, and shipped to the CSU Center for Environmental Medicine Analytical Laboratory for liquid chromatography-tandem mass spectrometry (LC-MS/MS) analysis using methods developed to detect PGD_2_, PGE_2_, and PGF_2_ (manuscript in preparation).

### PGE_2_ Detection After Low or High LET IR Under Normoxic or Hypoxic Conditions

HeLa cells were maintained in either normoxic (ambient) or 1% oxygen concentrations for 72 h after irradiation with low LET X-ray, either of two moderately high LET carbon ion beams (290 MeV/nucleon monoenergetic beam at 30 keV/μm, or 290 MeV/nucleon monoenergetic beam at 70 keV/μm), or a higher LET silicon ion beam (135 or 490 MeV/nucleon monoenergetic beam at 300 keV/μm). PGE_2_ in the supernatant media was detected by ELISA. Normoxic and hypoxic cells that did not receive IR served as controls. Pretreatment with 10 μM COX-1/COX-2 inhibitor (Indo) was the same as described above.

### Functional Assay for IR-Induced, PGE_2_-Stimulated Growth of Co-Cultured, Unirradiated Cells

Twenty-four hours prior to IR, 30,000 (no IR) or 100,000 (to be irradiated) HeLa cells were seeded into wells of six-well plates and incubated at 37°C in 5% CO_2_ air. Additionally, 1,000 or 1,500 HeLa cells were seeded into ThinCert transwell inserts with 1 μm pores (Greiner), placed into 10 cm dishes and incubated at 37°C in 5% CO_2_ in air. Once cells had attached, transwells were transferred into their corresponding wells. Control wells were prepared with equal numbers of cells in the transwells but no cells below. Twelve hours prior to IR, cells were treated with DDR inhibitors and/or Indo as above. Six-centimeter spread out Bragg Peak (SOBP) beams of moderately high LET carbon ions (290 MeV/nucleon, dose average LET of 50 keV/μm at the center of the SOBP) were generated at the Heavy Ion Medical Accelerator (HIMAC) facility of the National Institute of Radiological Sciences (NIRS), Chiba, Japan. The transwells for the irradiated plates were transferred to six-well holding plates immediately preceding irradiation and the media in the remaining wells was aspirated. Vertically oriented plates containing the cells were irradiated with 4 Gy carbon ion IR. Following irradiation, fresh media containing DDR inhibitor was added to the wells and the transwells, and the transwells were returned to their previous position. For the no IR controls, the media (in wells and transwells) was aspirated and fresh media containing DDR inhibitor was added and dishes were incubated for 3–6 days. On day 3 or day 6 post-irradiation, PGE_2_ concentrations in transwell media was analyzed by ELISA. On day 7, sufficient media was added to transwells to allow cell growth for two more days, and on day 9 post-irradiation, transwell cells were trypsinized, and resuspended in 500–1000 μL of PBS and counted using a Coulter Counter or Scepter cell counting device (EMD Millipore).

### Analysis of Apoptosis by Caspase 3/7 Cleavage and Annexin V Assays

Duplicate dishes were prepared for apoptosis assays as follows. Twenty-four hours prior to IR, 60,000 (no IR control) or 250,000 (to be irradiated), HeLa cells were seeded into wells of six-well plates and incubated at 37°C in 5% CO_2_ in air. Twelve hours prior to IR, cells were treated with DDR and/or COX-1/COX-2 inhibitors and irradiated in parallel as described in the previous section. Following irradiation cells were incubated for 72 h and subsequently assayed by flow cytometry for two apoptosis endpoints. Caspase 3/7 cleavage/activation was monitored by cleavage of a DEVD peptide substrate conjugated to Alexafluor 488 (Cell Event 3/7 Caspase Green Reagent, Life Technologies) as follows. Cells from non-irradiated and irradiated treatment groups were trypsinized, harvested, and combined with supernatants from each well (containing potential apoptotic cells), centrifuged at 1200 rpm for 5 min, and the media was removed by aspiration. Cells were suspended in 500 μL of fresh media containing 1 μL Cell Event reagent (4 μM final concentration) and incubated at 37°C in 5% CO_2_ in air for 15 min, then 500 uL of PBS was added to each sample and cells were analyzed on a BD FACSCaliber flow cytometer using 488 nm excitation and collecting fluorescent emissions with a 530/30 filter set. Gates were set using unstained and no treatment/no IR cells as negative control populations. Data represent the percent caspase-positive cells among 10,000 cells analyzed per sample. Data were acquired with CellQuest (Becton Dickinson) software, and analyzed using FloJo (Version 7.6.5, Tree Star Inc.) and Prism (Version 5.04, GraphPad) software.

Annexin V (AV) is a Ca^2+^-dependent phospholipid binding protein that binds with high affinity to phosphatidyl serine residues that have translocated to the outer leaflet of the plasma membrane as a result of upstream apoptotic signaling, representing an early marker of apoptosis. Propidium iodide (PI) is a cell impermeant DNA binding dye that will penetrate into cells with compromised (leaky) membranes indicative of cellular necrosis, representing late-stage apoptosis. It is possible to discriminate the early (AV only), middle (AV and PI double positive), and late (PI-positive only) stages of cell death by co-staining with AV and PI. Approximately 5 × 10^5^ cells (and supernatants containing potential apoptotic cells) were harvested from wells processed as above to generate cell pellets which were washed once in cold PBS, harvested by centrifugation, and suspended in 500 μL annexin binding buffer (10 mM HEPES, 140 mM NaCl, 2.5 mM CaCl_2_, pH 7.4) yielding cell concentrations of approximately 1 × 10^6^ cells/mL. Three microliters of Annexin V, Alexa Fluor 488^®^ conjugate (Life Technologies) and 150 μL of annexin binding buffer were aliquoted to flow cytometry tubes, and 150 μL of cell suspensions were added, samples were mixed, incubated at room temperature for 15 min, and an additional 300 μL of annexin binding buffer was added, samples were mixed, stored on ice, and analyzed on a BD FACSCaliber flow cytometer using 488 nm excitation and collecting fluorescent emissions for FL1 and FL2 parameters using 530/30 and 585/42 filter sets, respectively. Compensation was established using the single-stained samples for the +IR treatment group and quadrant gating was established to identify AV^−/^PI^−^ (apoptosis negative), AV^+^/PI^−^ (early apoptotic), AV^+^/PI^+^ (middle apoptotic), and AV^−^/PI^+^ (late apoptotic/necrotic) populations. Data represent the percentage of cells in each quadrant from 5,000–10,000 cells collected per sample, using data acquisition and analysis software as above.

### Clonogenic Cell Survival Assay

T25 flasks were seeded to ~20% confluence with cell lines to be tested, and incubated overnight. Cells were pretreated with DDR inhibitors at least 12 h prior to exposure to low or high LET IR. Cells were irradiated at ~50% confluence and allowed to recover for 30 min before they were trypsinized, harvested, suspended in fresh media, and counted using a Coulter Counter. Appropriate numbers of cells to yield ~100 colonies per 6 cm dish were suspended in fresh medium and distributed to three replicate dishes per treatment group. Forty-eight hours post irradiation, the media was aspirated and replaced with fresh media without drug. The cells were incubated for 8–11 days to allow colonies to develop. The dishes were stained with 0.5% (w/v) crystal violet in 70% methanol solution and the colonies were counted. Survival fractions were calculated and plotted using Excel and Prism software. We derived *p-*values for statistical analysis by using student’s *t*-tests.

## Results

### PGE_2_ Production and Cell Viability After IR Vary Among Cell Types and Are Regulated by the DDR

**Figure 2:**
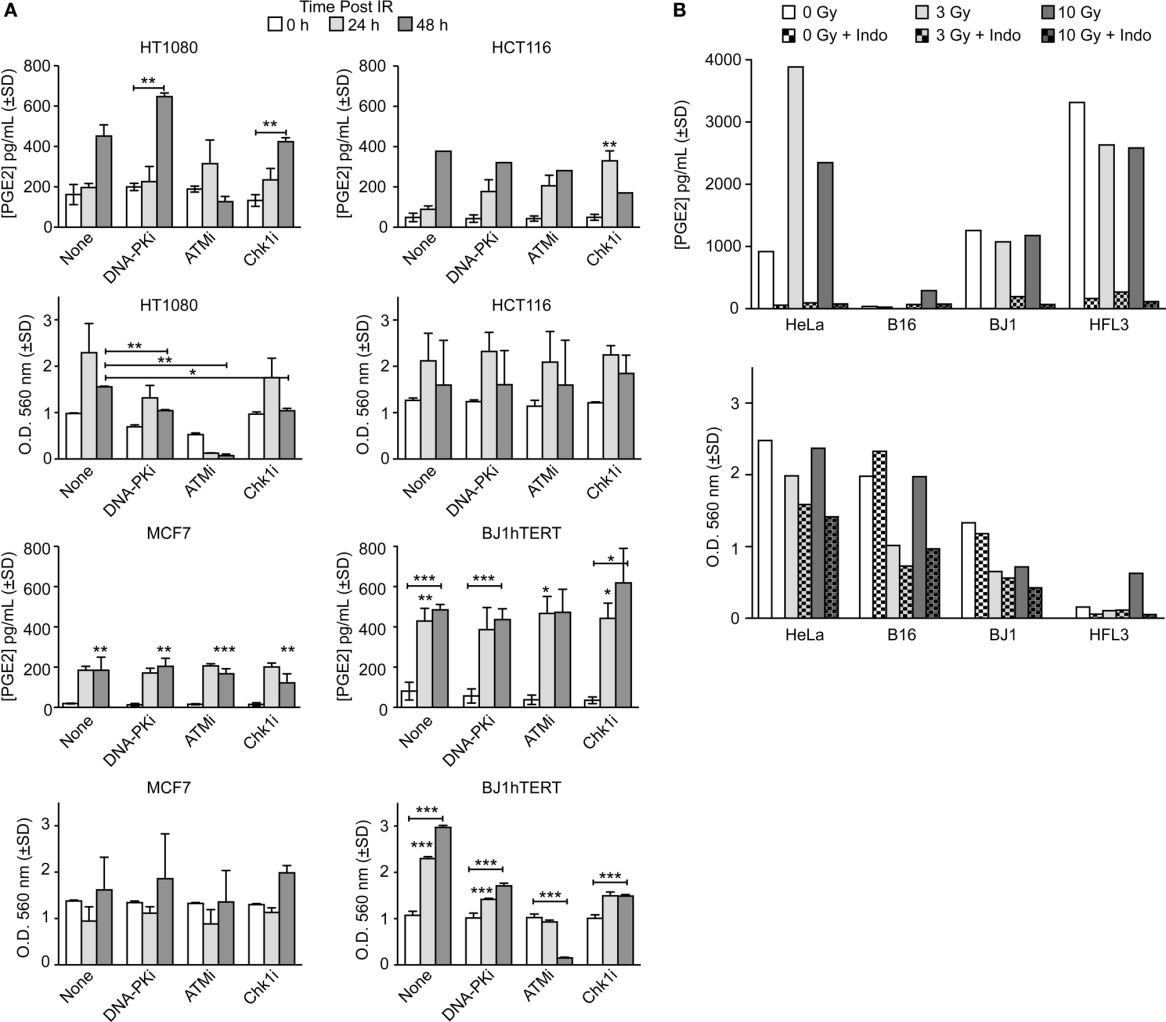
**PGE_2_ production and short-term cell viability/proliferation in response to γ-rays or X-rays in the presence or absence of DDR or COX inhibitors**. PGE_2_ and cell growth were measured by ELISA and SRB assay in response to **(A)** 10 Gy γ-rays or **(B)** 3 or 10 Gy X-rays in the presence or absence of indomethacin (Indo). Data are averages ±SD for two to four replicates per treatment group **(A)** or single determinations **(B)**. In this and all subsequent figures, statistical significance was determined by *t*-tests, * indicates *p* < 0.05, **p < 0.01, ***p < 0.001.

The goals of this study were to determine whether DDR inhibitors and/or oxygen alter PGE_2_ production and cell growth of irradiated cells in response to low and high LET ionizing radiation. We initially surveyed PGE_2_ production in five cancer cell lines (HT1080 fibrosarcoma, HCT116 colorectal carcinoma, MCF7 breast adenocarcinoma, HeLa cervical adenocarcinoma, and B16 mouse melanoma) and two normal cell lines (BJ1hTERT, hTERT-immortalized human foreskin fibroblasts, and HFL3 spontaneously immortalized human fetal lung cells) following exposure to low LET γ-rays or X-rays. In the absence of DDR inhibitors, PGE_2_ levels increased several fold 24 or 48 h after 10 Gy γ-rays with most cell lines, with statistically significant differences in MCF7 and BJ1hTERT, and trending toward significance in HT1080 (*p* = 0.06); HCT116 showed an approximately eightfold increase at 48 h, but significance could not be calculated because only a single determination was made at this time point (Figure 2A). In parallel with the PGE_2_ assays, we measured cell survival/proliferation by SRB assay and found significant increases in cell number 48 h after IR in HT1080 and BJ1hTERT, but not HCT116 nor MCF7 (Figure 2A). PGE_2_ effects on growth were previously observed at later times after IR (>5 days) (2) so the absence of a consistent early growth effect is not surprising. These PGE_2_ effects were observed at IR doses of 3–10 Gy, higher than the 2 Gy doses typically used in fractionated photon RT, but well within the range of doses used in hypofractionated stereotactic body RT. PGE_2_ production is likely proportional to levels of caspase activation (and apoptosis), but more studies are required to determine whether PGE_2_ production follows a standard dose–response or displays threshold effects. These data demonstrate that basal and IR-induced PGE_2_ levels, and early effects on survival/proliferation, vary among cell types.

DNA damage response inhibitors affected IR-induced PGE_2_ production that again varied among cell types. In control experiments, we confirmed that inhibitors of DNA-PK, ATM, and Chk1 reduced clonogenic survival (Figure S2 in Supplementary Material and data not shown). DNA-PKi slightly increased PGE_2_ production in HT1080 cells 48 h after IR, but the difference was not significant, and DNA-PKi had no effect on PGE_2_ production in HCT116, MCF7, or BJ1hTERT cells (Figure 2A). ATMi suppressed PGE_2_ levels in HT1080 cells 48 h post IR by ~1.5-fold, but ATMi did not affect PGE_2_ in other cell types. Chk1i significantly enhanced PGE_2_ production in HCT116, but other cell types showed no Chk1i effects. At 48 h after IR, ATMi dramatically decreased cell number (by ~20-fold) of both HT1080 and BJ1hTERT cells, whereas HCT116 and MCF7 cells were not affected. Because the SRB assay provides an estimate of survival/proliferation based on the amount of protein in attached cells (29), the sharp decrease in the number of attached HT1080 and BJ1hTERT cells with ATMi reflects massive cell death/detachment in response to the combined IR + ATMi treatment. Note that in both HT1080 and BJ1hTERT cells ATMi sharply reduced PGE_2_, but only HT1080 showed reduced cell numbers, suggesting that PGE_2_ is reduced by distinct mechanisms in these cell lines, with death and detachment preceding PGE_2_ production in HT1080, but not BJ1hTERT.

Prostaglandin E_2_ production depends on COX1–2 (Figure 1A) that can be inhibited with Indo. We next measured PGE_2_ and cell survival/proliferation with HeLa, B16, BJ1hTERT, and HFL3 cells 48 h after 3 or 10 Gy doses of X-rays in presence or absence of Indo (Figure 2B). With HeLa cells, PGE_2_ levels increased approximately fourfold with an X-ray dose of 3 Gy, and approximately twofold with 10 Gy. B16 cells produced very little PGE_2_ without IR or with a 3 Gy X-ray dose, but PGE_2_ increased eightfold at 10 Gy. HFL3 and BJ1hTERT cells had high basal levels of PGE_2_ that did not change substantially in response to X-rays. Uniformly, Indo dramatically suppressed PGE_2_ levels (>20-fold), including basal and X-ray induced levels in both cancer and normal cells (Figure 2B). These data concur with numerous studies showing that inflammatory prostaglandin production can be mitigated by COX1–2 inhibitors (30). The variable basal levels of PGE_2_ among cell lines may reflect differential expression or activation of PGE_2_ pathway proteins, including caspase 3, which may be activated when rapidly growing cells reach confluence and deplete growth media. There was a general trend toward decreased cell growth with increased IR dose. HeLa cells showed both stronger PGE_2_ induction with X-rays, and greater radioresistance than the other cell lines. Note that HFL3 cells showed poor viability/proliferation after IR, and high basal PGE_2_ levels, yet PGE_2_ was not induced with IR in these cells. These features may be specific to HFL3 cells or perhaps reflect general properties of fetal cells.

**Figure 3:**
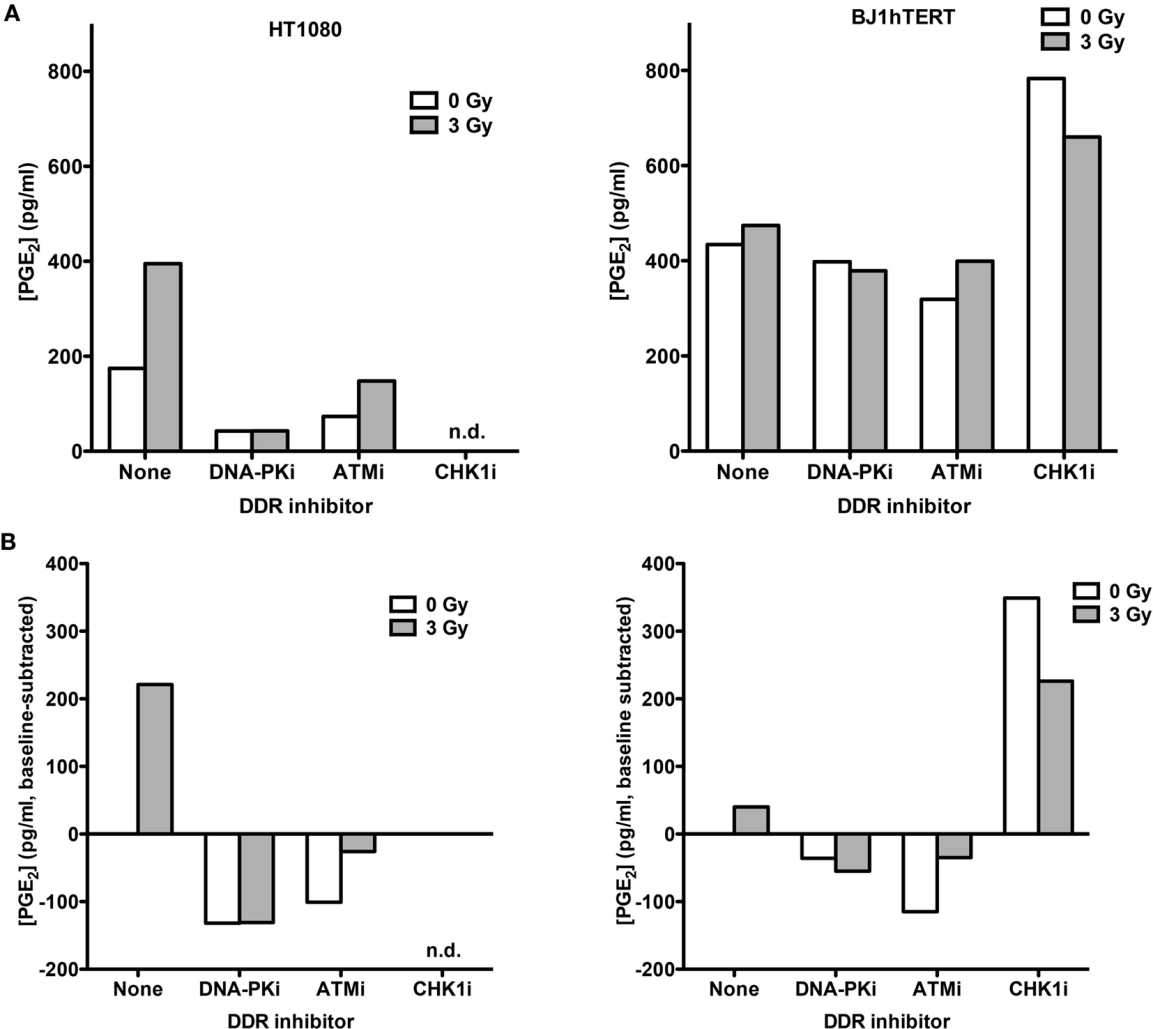
**PGE_2_ production in response to high LET IR ± DDR*i***. LC-MS/MS was used to quantify PGE_2_ levels in supernatants from HT1080 and BJ1hTERT cultures after irradiation with 3 Gy carbon ions (70 keV/μm). **(A)** Absolute PGE_2_ levels. **(B)** PGE_2_ levels were derived by subtracting the baseline values (no IR, no drug); n.d., not determined.

To determine if low and high LET IR produce similar PGE_2_ responses, absolute PGE_2_ levels in media from HT1080 and BJ1hTERT cultures were determined by LC-MS/MS in response to 3 Gy high LET (70 keV/μm) carbon ions (Figure 3). This alternate PGE_2_ assay helped us validate and expand on findings from the ELISA assay. In the absence of DDR inhibitors, HT1080 cells showed robust PGE_2_ induction with carbon ions, similar to the effect of low LET IR, and BJ1hTERT cells were unresponsive to both low and high LET IR (Figures 2 and 3). These results indicate that PGE_2_ production is stimulated by both low and high LET IR in a cell-type-dependent manner. The variation in absolute basal levels of PGE_2_ in BJ1hTERT cells measured by LC-MS/MS and ELISA may reflect different sensitivities of ELISA and LC-MS/MS assays.

Inhibition of DNA-PK and ATM decreased carbon ion-induction of PGE_2_ in HT1080 cells. The decrease in PGE_2_ with DNA-PKi after high LET contrasts with that seen with γ-rays. This difference in DNA-PKi effects with low and high LET IR could reflect DNA-PK’s dual role in damage signaling and DSB repair by NHEJ (Figure 1B), in particular, the shift from NHEJ-dominant repair of low LET IR damage to HR-dominant repair of high LET damage (31–33). By contrast, Chk1 inhibition markedly increased PGE_2_ in BJ1hTERT cells, with or without IR. The Chk1i effect in the absence of IR suggests that the cytotoxicity of Chk1i alone is sufficient to trigger Phoenix Rising. This Chk1i effect may reflect aberrant signaling to the p53-directed apoptotic cascade culminating in PGE_2_ production, since p53 is stabilized by Chk1 phosphorylation of several sites after DNA damage (34). Together the results indicate that PGE_2_ production following low or high LET IR can be enhanced or suppressed by inhibition of different pathways in the DDR network.

### IR-Induced PGE_2_ Stimulates Proliferation of Co-Cultured, Unirradiated Cells that can be Modulated by DDR and COX1–2 Inhibitors

**Figure 4:**
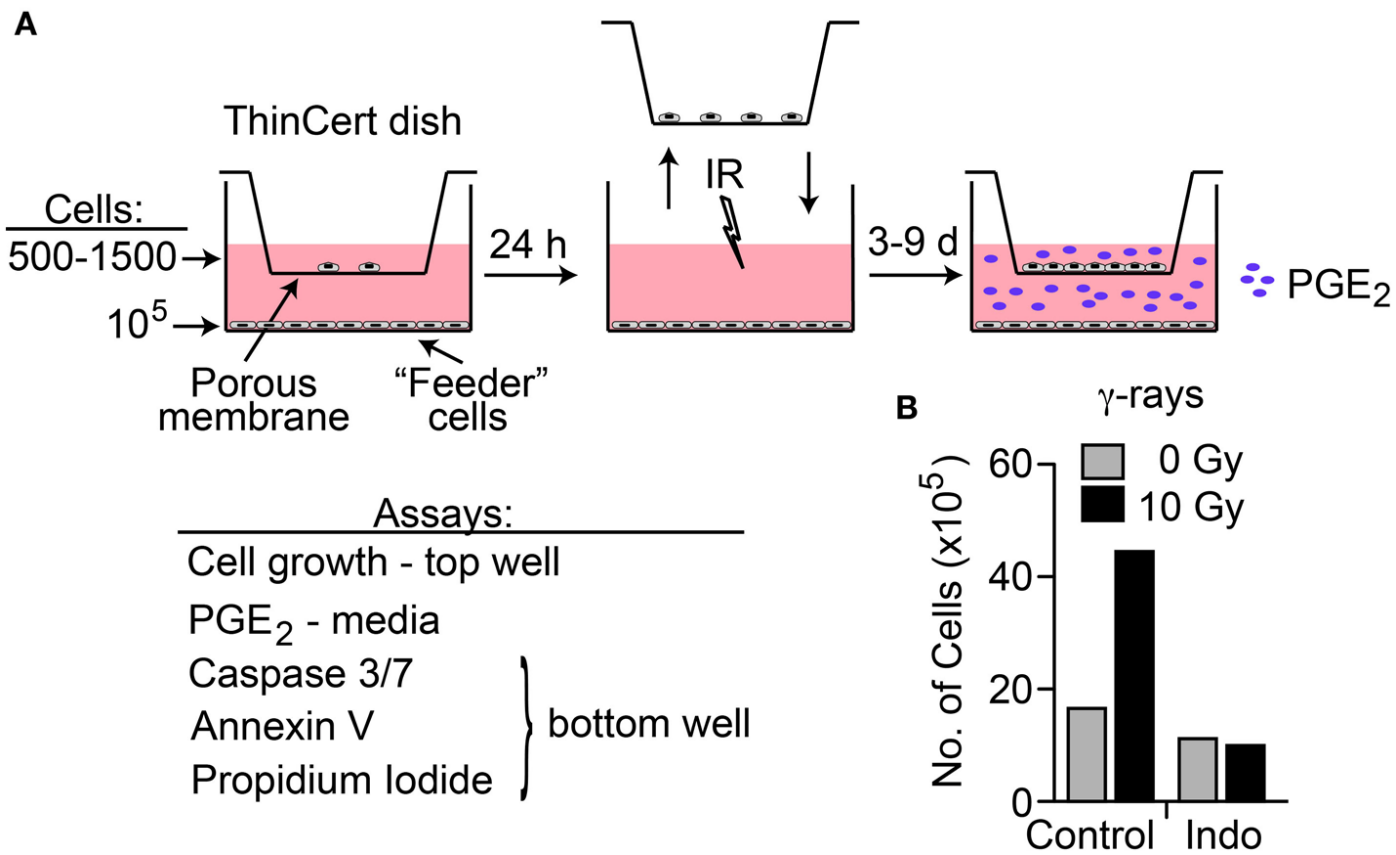
**(A)** Transwell multiple-endpoint assay system. Cells were seeded into the top (500–1,500 cells) and bottom (feeder, 100,000 cells) sections of the transwell system and incubated for 24 h. The top section was transferred to a separate dish while the bottom section was irradiated, and then replaced. PGE_2_ was measured in the media 3 and 6 days after IR; growth of unirradiated cells was determined 7–9 days after IR. Apoptotic endpoints (caspase 3/7 activation, Annexin V, and membrane changes by propidium iodide staining) were measured at 3, 6, or 9 days post IR in irradiated feeder cells. When used, DDR or cyclooxygenase inhibitors were present during the entire experiment. **(B)** HeLa cells assayed for growth stimulation using the transwell assay system after irradiation with 10 Gy low LET γ-rays. Indomethacin (Indo) inhibits COX1–2 and suppresses PGE_2_ production.

A transwell multiple-endpoint assay system was used to examine the effects of low and high LET IR on co-cultured irradiated and unirradiated cells (Figure 4A). The pore size of the transwell membranes allows free diffusion of small molecules, but cell migration is blocked. This system allows simultaneous analysis of PGE_2_ production, apoptosis, and cell growth as a key functional endpoint. We assessed the effects of radiation quality, DDR and COX1–2 inhibitors, and oxygen concentration on these end-points. We chose HeLa cells for these studies because the cell line survey showed that HeLa cells have low basal PGE_2_ levels, and relatively robust PGE_2_ production after IR that is blocked by cyclooxygenase inhibition. A preliminary test of cell proliferation of unirradiated co-cultured cells 7 days after “feeder” cells received 10 Gy γ-rays showed a 2.7-fold increase in growth compared to control with mock-irradiated feeder cells, and that COX1–2i suppressed this growth (Figure 4B). This result is similar to a prior report in which luciferase-expressing cancer cell growth was enhanced two- to fivefold when in direct contact with irradiated feeder cells (2). The transwell system eliminates any influence of cell-to-cell contact, measuring only the growth effects of diffusible factors through the transwell membrane.

**Figure 5:**
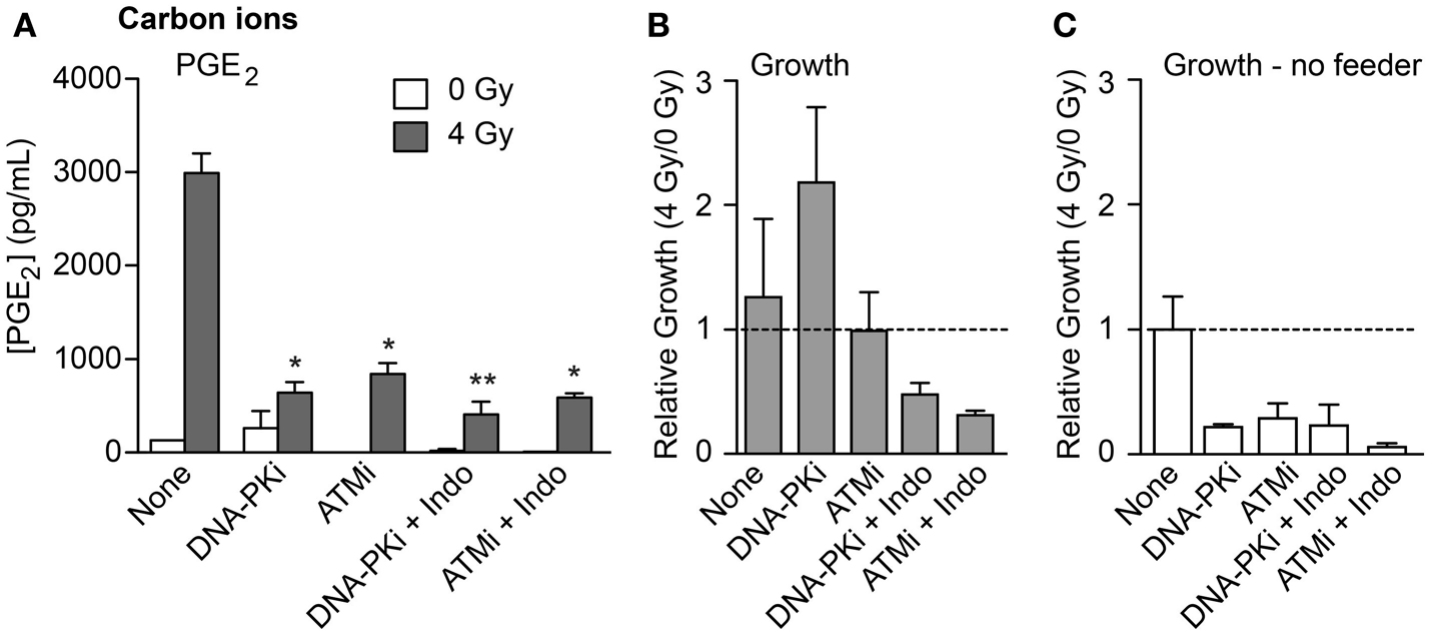
**PGE_2_ production and stimulated proliferation of unirradiated HeLa cells in response to high LET carbon ions**. **(A)** ELISA measurements from media in transwells containing HeLa cells 6 days after 0 or 4 Gy carbon ion irradiation. **(B)** Relative growth of unirradiated cells in transwells with or without DDR inhibitors or indomethacin, 6 days after irradiation of feeder cells. **(C)** As in **(B)** but without feeder cells. Data are averages (±SEM) for two determinations per condition.

To investigate whether high LET IR would elicit similar growth effects on co-cultured unirradiated cells, we used the transwell system to monitor PGE_2_ production and cell growth in response to 70 keV/μm carbon ions. We also tested whether DDR inhibitors and/or the COX1–2 inhibitor Indo would modulate growth stimulation. PGE_2_ production and growth of unirradiated HeLa cells was measured 6 days after IR. PGE_2_ levels increased >20-fold in response to IR in the absence of inhibitors (Figure 5A), similar to the increase seen with X-rays (Figure 2B). Both DNA-PKi and ATMi significantly decreased IR-induced PGE_2_ levels (approximately fourfold), and these were further reduced by approximately twofold by Indo (Figure 5A), although the latter differences were not statistically significant. Growth of unirradiated co-cultured HeLa cells was also monitored 6 days after IR. At this time point, moderate effects on growth were observed in the absence of DDR inhibitors, DNA-PKi increased growth but ATMi had no effect (Figure 5B). The increased growth with DNA-PKi does not appear to correlate with the lower PGE_2_ level, but we note that PGE_2_ levels were approximately twofold higher with DNA-PKi than untreated cells 3 days after IR (data not shown); the growth stimulation seen 6 days after IR may reflect this early burst of PGE_2_, and the higher PGE_2_ levels without DNA-PKi 6 days after IR would enhance growth at later times. The increased growth with DNA-PKi relative to untreated cells was dependent on feeder cells; no growth stimulation occurred without feeder cells (Figure 5C). As expected, blocking PGE_2_ production with Indo (Figure 5A) also blocked growth stimulation (Figures 5A,B). As noted above, inhibiting DNA-PKcs or ATM radiosensitized cells (Figure S2 in Supplementary Material); thus, the differences in PGE_2_ and growth effects with DNA-PKi and ATMi cannot be traced to differential degrees of cell killing, suggesting that inhibition of different DDR pathways can differentially affect Phoenix Rising and death pathway choice.

**Figure 6:**
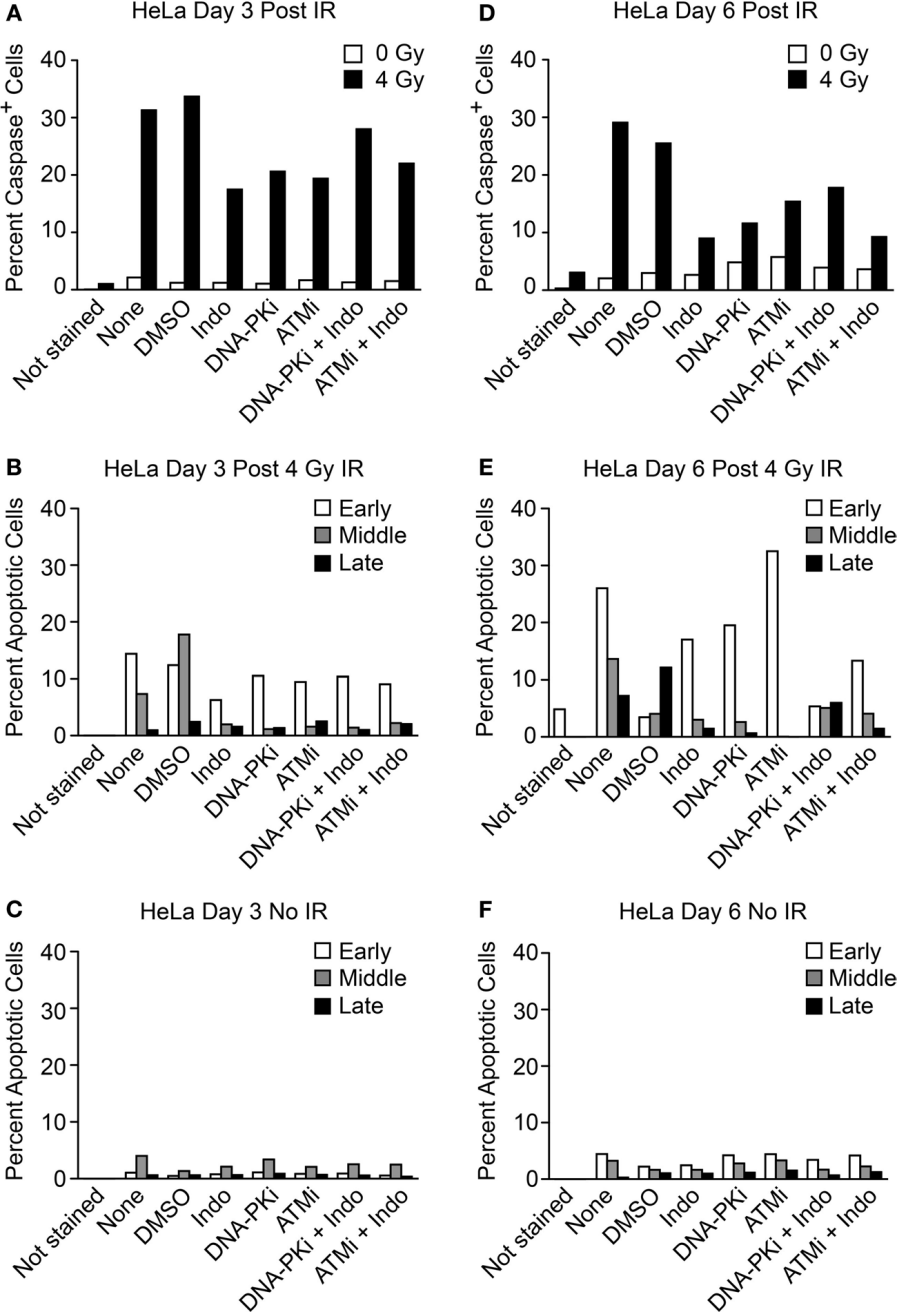
**Flow cytometric analysis of apoptotic phenotypes of feeder cells (from Figure 5) irradiated with high LET carbon ion IR**. Duplicate plates were irradiated in parallel for assays 3 days post IR. Feeder cells co-cultured with transwell inserts were assayed 6 days post IR. Each sample was split at the time of harvest for two assay endpoints. Caspase cleavage and activation was assayed in live cells **(A)** 3 or **(D)** 6 days post IR using a fluorescent caspase 3/7 peptide fragment. Annexin V/propidium iodide co-staining of fixed cells discriminates between early, mid, or late apoptotic stages and was assayed **(B)** 3 or **(E)** 6 days post IR. No IR controls for the annexin V/PI assay **(C)** 3 or **(F)** 6 days post IR; ~10,000 cells were interrogated per determination. Unstained cells served as negative controls.

The transwell assay system is versatile in that in addition to PGE_2_ and growth, simultaneous measures of apoptotic stages can be determined with the same cultures, including early apoptosis (caspase cleavage, detected with a cell-permeable caspase 3/7 DEVD peptide conjugated to a quenched fluorophore, which becomes fluorescent upon peptide cleavage), mid-apoptosis (Annexin V staining), and late apoptosis (revealed as gross membrane changes with propidium iodide staining). These endpoints were measured 3 and 6 days after high LET IR by flow cytometry (Figure 6). Minimal caspase activation was detected in unirradiated controls, and ~30% of cells activated caspase 3 and 6 days after IR. DDRi and Indo moderately suppressed caspase activation at both time points. Caspase is activated upstream of arachidonic acid that is processed by the Indo target, COX1–2, in the Phoenix Rising pathway (Figure 1A). This raises the possibility of a caspase–COX1–2 feedback loop, although the present experiments cannot rule out off target effects of Indo. Strikingly, ATMi appeared to completely block progression to later apoptotic stages; to a lesser extent DNA-PKi and Indo also suppressed progression to later apoptotic stages. Since ATMi is a strong radiosensitizer, the lack of progression to late apoptosis indicates that cells are shunted to one or more alternative death or senescence pathways.

### Robust Phoenix Rising Responses in Cell Lines Derived from Fresh Tumor Tissue

Cell lines freshly derived from patient tumor tissue have emerged as important models for cancer cell biology studies (28, 35–37). We chose HNSCC-derived cell lines because head and neck cancers are treated with low and high LET RT. Low passage tumor cell lines were tested within 3 months of culture expansion. Two cell lines, CUHN036 and CUHN065, senesced or grew too slowly to study. Two others, CUHN013 and CUHN067, were reproductively robust and viable throughout the course of the experiments. CUHN013 is from a moderately focally keratinizing submental mass tumor. CUHN067 is from a base of tongue tumor that displayed extensive perineural invasion. Radiosensitivity was evaluated by clonogenic survival after X-rays or a clinical, 6 cm SOBP, high LET (~50 keV/μm) carbon ion beam. CUHN013 was more radiosensitive than CUHN067 to both X-rays and carbon ions, and both cell lines showed typical carbon ion RBEs of ~2–3 (Figure S3B in Supplementary Material).

**Figure 7:**
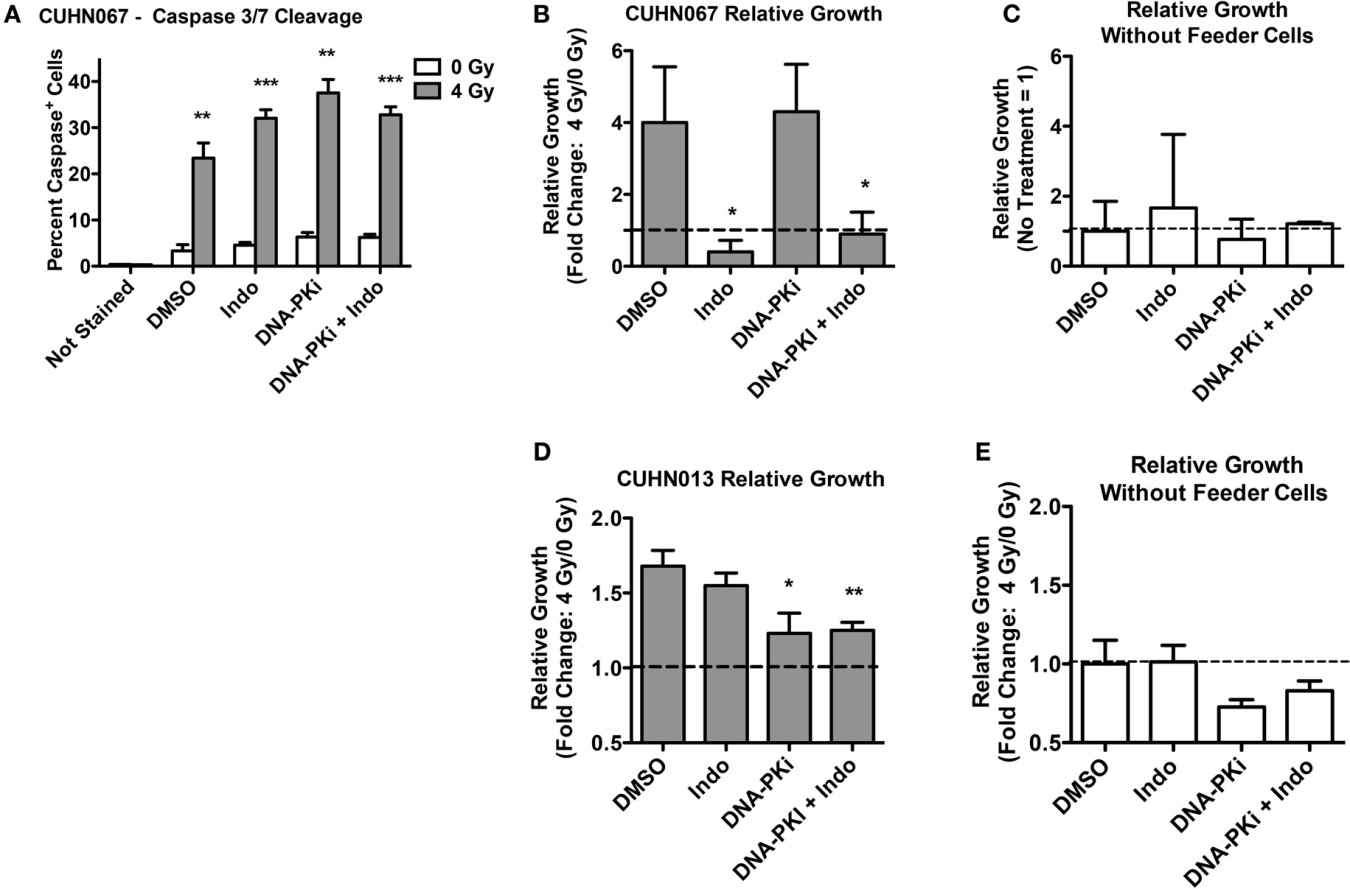
**Caspase activation and relative growth in two HNSCC cell lines after exposure to 4 Gy high LET IR**. **(A)** Caspase cleavage and activation was assayed in live cells 48 h post IR. **(B,D)** Relative growth of primary tumor cells from transwells 6 days post IR with or **(C,E)** without irradiated feeder cells. Data represent the averages and SDs for three replicate measurements per determination.

Doses of 4 Gy SOBP carbon ions significantly increased caspase 3/7 activation compared to mock-irradiated controls, and DDRi and Indo had little effect on this endpoint (Figure 7A). These results demonstrated that the initiating event of Phoenix Rising was functioning in these clinically relevant models. As with HeLa, in transwell experiments the CUHN cell lines showed moderate to strong growth stimulation of unirradiated cells that was dependent on irradiated feeder cells (Figures 7B–E). Neither CUHN cell line showed significant alterations in growth with DDRi or Indo. These early (caspase activation), late (growth stimulation, suppressible with Indo) Phoenix Rising markers indicate that Phoenix Rising is functioning in CUHN067. By contrast, CUHN013 displayed the early caspase marker and modest, but statistically significant (*p* < 0.01) growth stimulation, but Indo failed to suppress this growth, suggesting a late-stage defect. The distinct phenotypes of the two CUHN cell lines, in radiosensitivity and growth responses with or without inhibition of DNA-PK or COX1–2, highlight the challenges associated with targeting DDR and Phoenix Rising pathways for precision medicine.

### Oxygen Concentration and LET Attenuate PGE_2_ Production After Exposure to Carbon or Silicon Ion Beams

**Figure 8:**
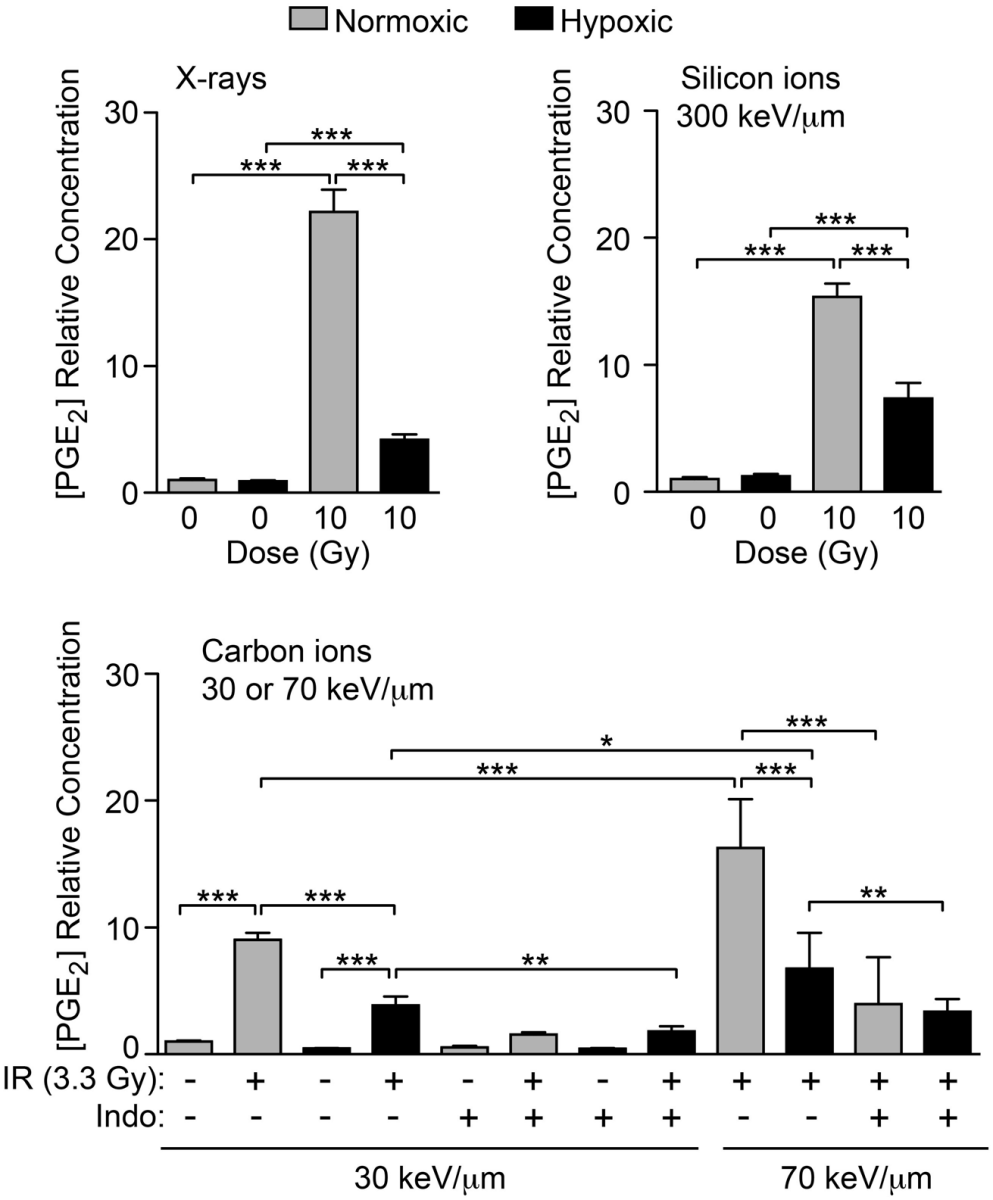
**IR-induced PGE2 production is suppressed by hypoxia**. PGE_2_ levels were determined by ELISA 3 days after X-ray, silicon ion, or carbon ion radiation at indicated LET and doses. Cells were incubated under normoxic, or hypoxic (1% O2) conditions. Data represent the averages and SDs for nine replicate measurements per determination.

COX1 and COX2 require sufficient oxygen to function efficiently. A number of tumor types are naturally hypoxic and/or have hypoxic regions. We tested the hypothesis that hypoxic cells will produce less PGE_2_ after IR than normoxic cells due to impaired COX1–2 function (Figure 1A). HeLa cells were incubated under normal conditions (5% CO_2_ in air, ~20% oxygen), or hypoxic conditions (1% oxygen) for 72 h after IR and PGE_2_ production was determined by ELISA. Normoxic and hypoxic cultures that did not receive IR served as controls. As above, 10 Gy X-irradiation increased PGE_2_ production by ~20-fold under normoxic conditions, but this was significantly reduced (to less than fivefold) under hypoxic conditions (Figure 8). Oxygen concentration had no effect on PGE_2_ levels without IR. We next tested the effects of oxygen on particle radiation at three LET values, 30 keV/μm carbon ion, 70 keV/μm carbon ion, and 300 keV/μm silicon ions. In all cases, hypoxia markedly reduced PGE_2_ production, and with 30 or 70 keV/μm carbon ions, treatment with Indo further reduced PGE_2_ production, in both normoxic and hypoxic treatment groups (Figure 8). Interestingly, 70 keV/μm carbon ions consistently caused approximately two- to threefold greater induction of PGE_2_ than 30 keV/μm carbon ions under normoxic or hypoxic conditions, and in the presence or absence of Indo. While this suggests an LET dependence for PGE_2_ production, this is questionable given that PGE_2_ induction was greatest with low LET X-rays, and that the highest LET radiation (300 keV/μm silicon ions) induced PGE_2_ no more than 70 keV/μm carbon ions; this parallels earlier findings that RBE peaks at 150–200 keV/μm. Further studies are required to determine whether LET dependence follows a complex pattern (e.g., plateaus above a certain LET), and whether there are signaling differences with photons vs. particle radiation that account for the high level of PGE_2_ induction with X-rays. Our results clearly indicate that, regardless of radiation quality, PGE_2_ production is very sensitive to oxygen concentration. Thus, hypoxic tumor regions are unlikely to contribute to tumor repopulation via Phoenix Rising.

## Discussion

The Phoenix Rising pathway correlates with tumor repopulation *in vitro* and *in vivo* (27, 38, 39). The present study revealed several important features of IR-induced PGE_2_ responses of normal and cancer cells. IR-induced, caspase 3-dependent, PGE_2_ production (whether associated with apoptosis or other death pathways), is a common response of irradiated tumor cells, and some normal cells. PGE_2_ levels generally enhanced growth of neighboring viable cells. Such growth, if unchecked, could spawn cells with high mutation rates, through replication of damaged genomes, as well as by an alternative mechanism activated in cells with moderately active caspase 3, that induces genome instability and carcinogenesis (40). Such small- and large-scale genetic change can drive rapid evolution of tumors, converting a local problem into lethal metastases.

Here, we focused on PGE_2_ production, cell viability, and proliferation of irradiated and unirradiated cells in response to signaling factor(s) from co-cultured irradiated, apoptotic “feeder” cells; the modulation of these responses by DDR and COX1–2 inhibition and by varying oxygen concentration; and the effects of radiation quality. Interconnected DDR and growth pathways coordinate responses radiation and chemotherapy-induced DNA injury and conspire with programed cell death pathways to determine cell fate (Figure 1B). In this study, we found that PGE_2_ production and growth responses varied among cancer and normal cell lines, including cell lines freshly derived from patient tumors.

Although DDR inhibition enhanced radiosensitivity (Figure S2 in Supplementary Material), this did not correlate with a single pattern (increase or decrease) in PGE_2_ production. Inherent radiosensitivity, and DDRi effects on radiosensitivity, varies widely among different cell types (4, 41) as observed here (Figures S2 and S3 in Supplementary Material). In general, the effects of radiation on the various endpoints (caspase activation, PGE_2_ production, growth) were consistent across all radiation types tested. This suggests that targeting PGE_2_-driven accelerated tumor repopulation may be an effective adjunct to both photon and particle radiotherapies. A fairly common response of ATMi and DNA-PKi was suppression of PGE2 production. A plausible explanation for this effect stems from the observations that ATM and DNA-PK activate the NFκB transcription factor in response to IR and other genotoxins (42–45), and COX-2, the upstream regulator of PGE2, is one of many NFκB targets (46). Because of the extensive crosstalk between DDR and growth factor networks, considerably more effort is required to define the multitude of tumor type responses to genetic and chemotherapeutic manipulation of DDR/growth regulatory factors. Phenotypic patterns emerging from such studies will guide mechanistic understanding of these various pathways, and may lead to rapid, inexpensive screening tools that will inform RT practice.

One facet that is consistent throughout our study and several others (2, 27, 47) is caspase activation and PGE*_*2*_* production. This is important because caspase status in tumors correlates with patient survival: patients with caspase 3-positive tumors do not survive as long as those with caspase 3-negaitve tumors (2, 48, 49). In addition, a recent study showed that low doses of radiation cause partial caspase 3 activation that leads to genome instability *in vitro* and *in vivo* through the generation of persistent DNA strand breaks (40). Another recent study demonstrated that caspase 3 defects created by shRNA, dominant negative gene expression, or gene deletion suppressed tumor growth *in vitro* and *in vivo* (47). Together, these findings implicate caspase 3 as a promising target to improve chemotherapy or RT outcomes.

COX1 and COX2 are also promising targets to enhance cancer therapy. The present study and others (2, 40, 47, 50) demonstrate that PGE_2_ production and stimulated growth can be effectively suppressed by Indo and other COX1 and COX2 inhibitors. Non-steroidal anti-inflammatory compounds (NSAIDS) effectively target cyclooxygenase enzymes and are generally safe. Our study focused on Indo, a pan-COX1–2 inhibitor; other well-characterized examples are Naproxen and Ibuprofen, which are widely used and well tolerated. Selective COX2 inhibitors, such as Celecoxib, have been marketed but many have been withdrawn due to increased risk of myocardial infarction; it is possible that such risk is minimal for short-term courses during cancer therapy. A body of literature is beginning to emerge that describes tests of cyclooxygenase inhibitors in combination with RT or chemotherapy. In one study, Celecoxib delivered between rounds of gemcitabine and cisplatin substantially suppressed bladder urothelial carcinoma xenograft regrowth, and enhanced the chemotherapeutic response in xenografts from a chemoresistant patient (7). An earlier study of LNCaP-COX2 mouse xenografts showed that topical application of the NSAID Diclofenac significantly reduced tumor growth in combination with 3 Gy IR (51).

Finally, we demonstrate that PGE_2_ production in response to IR is highly sensitive to oxygen concentration, including low and high LET radiation. Hypoxic regions of tumors are typically resistant to low LET IR. By contrast, high LET IR has a minimal oxygen enhancement ratio and, therefore, has greater efficacy than photons against these hypoxic regions (52). PGE_2_-stimulated tumor repopulation may not be a critical issue to consider during treatment planning for hypoxic regions of tumors. We stand at a new frontier on the path toward personalized precision medicine. As basic scientists and clinicians look to improve the efficacy and safety of cancer treatment in the future, it will be important to develop techniques for rapid, accurate detection of Phoenix Rising biomarkers to personalize patient care. Preventing accelerated tumor repopulation during RT and chemotherapy will improve local control and reduce the likelihood that early stage cancer will progress to more dangerous invasive and metastatic stages.

## Author Contributions

CA, WT, NS, YF, RO, AF, MD, and JN conceived and designed experiments; CA, WT, NS, CS, FN, MK, and AF conducted experiments; CA, WT, NS, FN, MK, AF, MD, and JN participated in data analysis; CA, WT, SK, AJ, and JN wrote the manuscript; all authors participated in critical revision.

## Conflict of Interest Statement

The authors declare that the research was conducted in the absence of any commercial or financial relationships that could be construed as a potential conflict of interest.
