# Supplementary material for: DNA Damage Response Proteins and Oxygen Modulate Prostaglandin E2 Growth Factor Release in Response to Low and High LET Ionizing Radiation
Source: Front Oncol. 2015 Dec 7;5:260. doi: 10.3389/fonc.2015.00260 (PMC4670845; doi:10.3389/fonc.2015.00260)
Supplement: Supplementary file 1 [file Presentation_1.PDF]

Supplementary Material

DNA Damage Response Proteins and Oxygen Modulate Prostaglandin E_2 Growth Factor Release in Response to Low and High LET Ionizing Radiation

Christopher P. Allen, Walter Tinganelli, Neelam Sharma, Jingyi Nie, Cory Sicard, Francesco Natale, Maurice King III, Steven B. Keysar, Antonio Jimeno, Yoshiya Furusawa, Ryuichi Okayasu, Akira Fujimori, Marco Durante, and Jac A. Nickoloff*

* Corresponding Author: J.Nickoloff@colostate.edu

Supplementary Figures

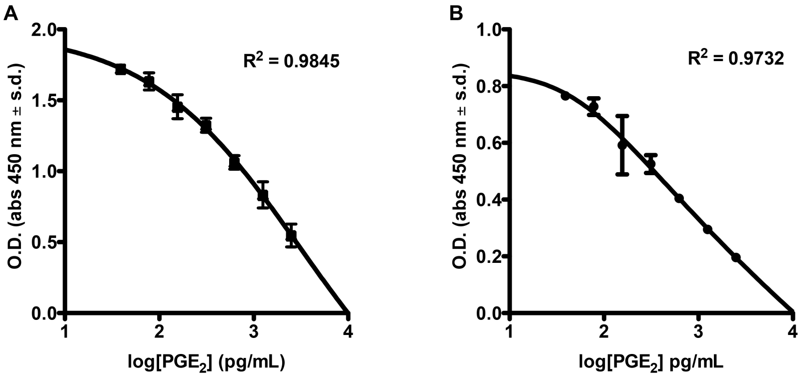

Supplementary Figure 1. (A) Curve fit used to derive the data shown in Figure 2A; (B) curve fit for Figure 2B. Asymmetric 5-parameter logistic non-linear regressions were fit to ELISA-based O.D. measurements from 2-fold dilutions of a pure PGE_2 standard. Data represent the average (±SD) for 2 replicates per determination.

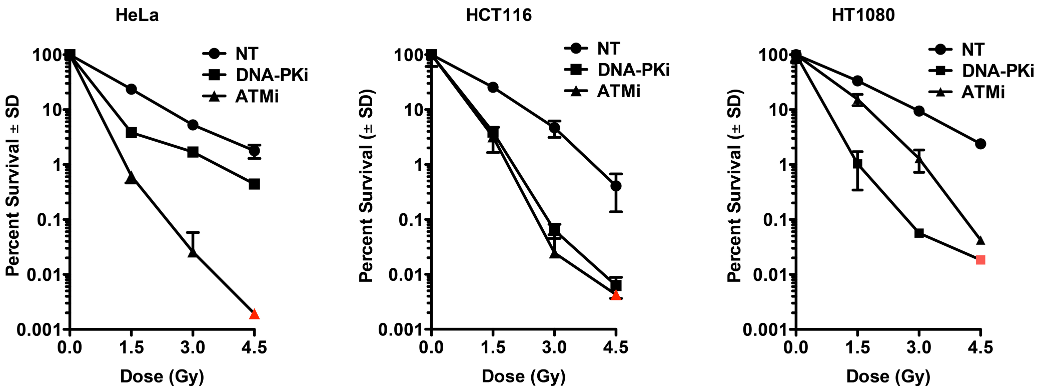

Supplementary Figure 2. IR survival curves. HeLa, HCT116, and HT1080 cells were irradiated with 1.5, 3 and 4.5 Gy X-rays with or without 10 mM DNA-PKi (NU7026) or ATMi (KU55933). Data are averages (±SD) for three replicate dishes per determination. Red data points represent maximum survival values, calculated as 1 colony per 160,000 HeLa cells, 1 colony per 215,053 HCT116 cells, or 1 colony per 114,000 HT1080 cells.

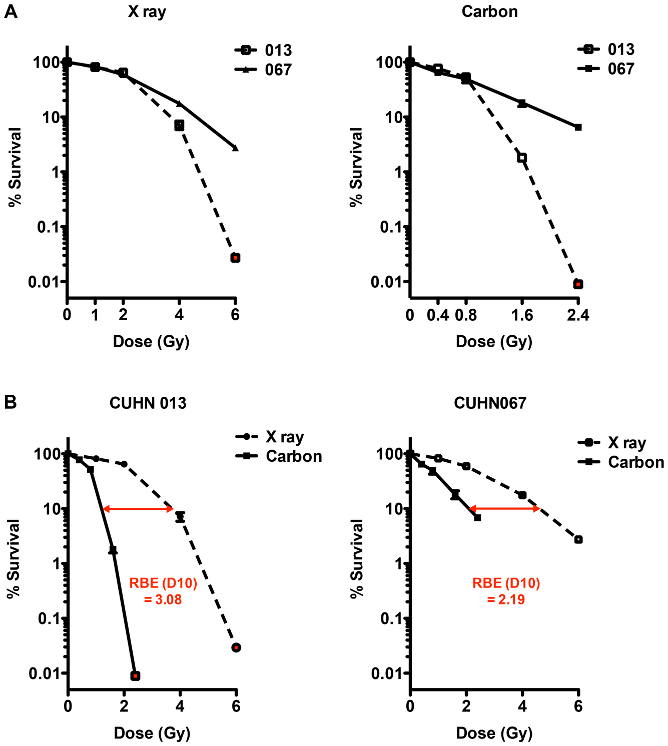

Supplementary Figure 3. (A) Radiation survival curves and (B) RBE determinations for CUHN013 and CUHN067 HNSCC primary tumor cell lines irradiated with X-rays or 70 keV/µm carbon ion beams. Red symbols are calculated maximum values based on 1 colony from 15,000 (X-ray) or 40,000 (carbon) cells plated per dish. Data represent the averages (±SD) for 3-6 replicates per determination.
